# A Case of Septic Portal Vein Thrombosis in a 71-Year-Old Female

**DOI:** 10.7759/cureus.27256

**Published:** 2022-07-25

**Authors:** Juliana S Ali, Renée H Kinden, Jason G Emsley

**Affiliations:** 1 Emergency Medicine, Dalhousie University, Halifax, CAN

**Keywords:** septic thrombophlebitis, pylephlebitis, portal vein thrombosis, septic pvt, pvt

## Abstract

Portal vein thrombosis (PVT) is a relatively rare condition that is characterized by partial or complete occlusion of the portal vein. The most common risk factors for developing PVT are a result of a low intra-hepatic vein flow or pro-thrombotic states, including underlying liver disease, coagulopathies, infection, and malignancy. Patients with PVT can present asymptomatically, while others are in profound shock. Clinical manifestations vary based on the location of the thrombus, degree of occlusion, and if it has become infected. Although an uncommon source of sepsis in the emergency department (ED), maintaining a high degree of clinical suspicion for septic PVT is critical as there are additional treatment considerations apart from early antibiotic therapy as in general sepsis. The following case report focuses on a 71-year-old woman with a septic PVT who presented to the ED with fever and hypotension in the absence of known risk factors. Current management guidelines and evidence regarding treatment strategies for septic PVT are also discussed in further detail.

## Introduction

The portal vein is formed by the confluence of the superior mesenteric and splenic veins and serves to drain the abdominal alimentary tract as well as the spleen, pancreas, and gallbladder. Portal vein thrombosis (PVT) occurs due to the occlusion of the intrahepatic portal vein branches, the main portal vein, or a combination of both; it may also propagate in a retrograde fashion to involve the superior mesenteric and splenic veins. Such thrombi are largely caused by low-flow states within the portal vein or hypercoagulable states [1]. Risk factors that may predispose patients to PVT include cirrhosis, malignancy, infection, surgery, myeloproliferative neoplasms, inflammatory bowel disease, and the use of oral contraceptives [2].

PVT can be classified as chronic or acute based on clinical presentation and imaging studies. Acute PVT often presents with sudden onset severe abdominal pain, or pain that may progress over the course of a few days, along with diarrhea and abdominal distension. In patients with cirrhosis, there may be a worsening of ascites or extant varices. Chronic PVT may be largely asymptomatic except for potential manifestations of portal hypertension complications, including persistent ascites or varices. Radiographically, chronic thrombus is characterized by the presence of venous collaterals [3]. Unfortunately, asymptomatic or mild PVT presentations and a lack of prior imaging can create diagnostic uncertainty when classifying chronicity.

Septic PVT, also known as pylephlebitis, is a suppurative thrombosis of the portal vein secondary to infection. The most common cause is diverticulitis, followed by appendicitis [4]. Patients often present with fever, abdominal pain, and leukocytosis. Liver function tests may be normal or elevated [4-5]. PVT, and particularly septic PVT, are medical emergencies with the latter having a mortality rate of 25% [4]. Even with early recognition and treatment, there is a high risk of intestinal infarction and multi-organ failure.

## Case presentation

Our patient was a 71-year-old female who presented to a tertiary care emergency department (ED) with a three-day history of fever, chills, general malaise, and constant upper abdominal pain radiating into her lower chest and right flank. She endorsed several bouts of nausea and non-bloody, non-bilious vomiting, as well as constipation. 

On initial exam, the patient appeared uncomfortable and was slightly confused despite remaining oriented to person, place, and time. The patient was febrile, tachycardic, and tachypneic but maintained normal oxygen saturation on room air (Table 1). The patient’s triage blood pressure was originally normotensive, however, she became progressively hypotensive despite intravenous fluid resuscitation. Her abdomen was diffusely tender to palpation, with maximal discomfort and guarding in the upper quadrants. She also had right-sided costovertebral angle tenderness. Her cardiovascular and respiratory exams were unremarkable.

**Table 1:** Patient’s vital signs on arrival at the emergency department Temp: Temperature (Tympanic); HR: Heart rate; RR: Respiratory rate; BP: Blood pressure; O2: Oxygen saturation.

Temp	HR	RR	BP	O2
39.2 °C	130; regular	28	118/67	97%

Her past medical history was significant for polymyalgia rheumatica, type 2 diabetes, hypertension, psoriasis, and chronic obstructive pulmonary disease (COPD). Her home medications included prednisone, naproxen, metformin, gliclazide, candesartan, hydrochlorothiazide, salbutamol, and fluticasone. She had no history of recent surgery and denied sick contacts.

Our differential diagnosis included urosepsis secondary to a renal stone, aortic dissection, mesenteric ischemia, perforated viscus, cholangitis, renal artery thrombosis, and other vascular causes. The patient’s febrile presentation with biochemical evidence of acute inflammation with poor perfusion led to a working diagnosis of sepsis and broad-spectrum antibiotics (piperacillin-tazobactam) were empirically initiated. Initial investigations were notable for a leukocytosis, elevated lactate, an acute kidney injury, and hepatic transaminitis in the context of a normal anion gap metabolic acidosis (Table 2). Blood cultures were drawn and sent. Her urinalysis and chest X-ray were unremarkable.

**Table 2:** Initial lab results in the emergency department RBC: Red blood cells; WBC: White blood cells; GGT: Gamma-glutamyl transferase; AST: Aspartate aminotransferase; ALT: Alanine transaminase; ALP: Alkaline phosphatase; PCO2: Partial pressure of carbon dioxide; HCO3: Bicarbonate; O2: Oxygen.

Test	Result	Value	Reference Range
Hemoglobin	132	Normal	120-160 g/L
RBC	4.56	Normal	3.80-5.80 x10^12^/L
WBC	15.44	High	4.50-11.00 x10^9^/L
Platelets	240	Normal	150-350 x10^9^/L
Creatinine	149	High	49-90 μmol/L
Bilirubin Direct	9.6	High	0.0-8.5 μmol/L
Bilirubin Total	12.5	Normal	0.0-20.4 μmol/L
GGT	83	High	0-49 U/L
AST	138	High	5-45 U/L
ALT	171	High	0-44 U/L
ALP	78	Normal	38-150 U/L
Albumin	26	Low	35-50 g/L
Sodium	129	Low	136-145 mmol/L
Potassium	4.4	Normal	3.4-5.0 mmol/L
Ionized Calcium	1.18	Normal	1.15-1.27 mmol/L
pH	7.38	Normal	7.32-7.43
PCO2	33	Low	38-50 mmHg
HCO3	20	Low	22-29 mmol/L
O2 Content	14.0	Normal	11.6-15.6 mL/dL
Lactate	3.7	High	0.5-2.2 mmol/L
Lipase	29	Normal	8-78 U/L

As the patient became progressively hypotensive, the CT scan was appropriately delayed for resuscitation. Considering the minimal response to intravenous fluids, the patient was started on vasopressor support and was given stress dosing of methylprednisolone to maintain perfusion. Once hemodynamically stable, the patient was sent for an urgent contrast-enhanced CT scan of the chest, abdomen, and pelvis to rule out potential sources of sepsis.

**Figure 1:**
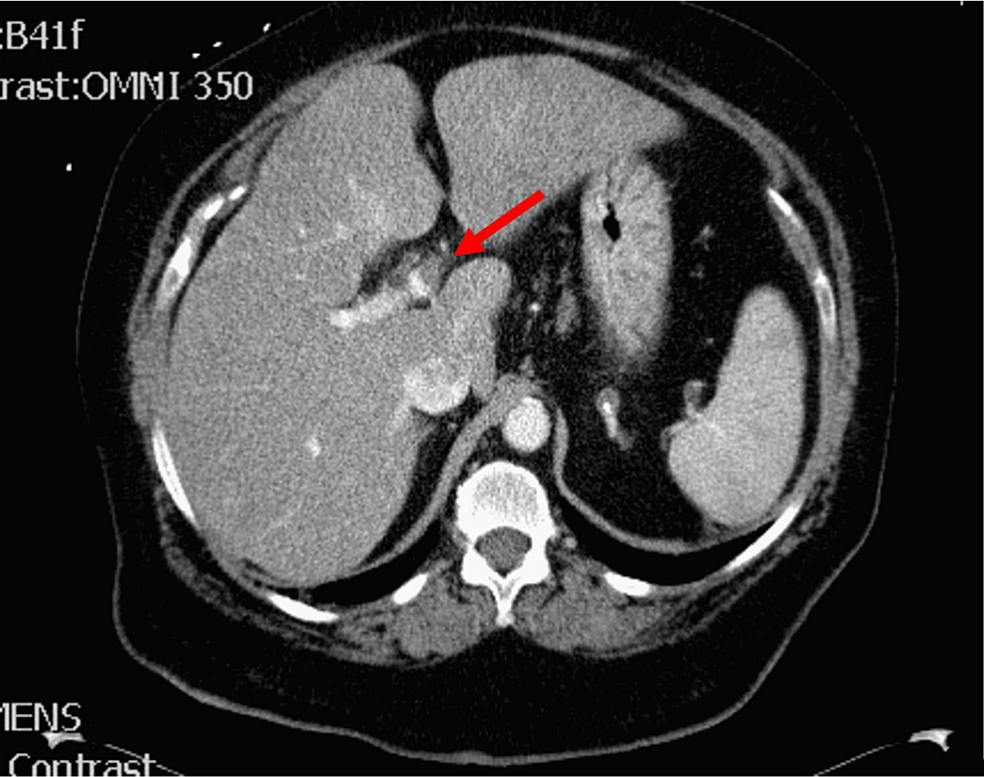
CT scan showing a bland thrombus within the left portal vein with subsequent mild heterogenous enhancement of the left hepatic lobe

The patient’s CT scan was notable for a significant thrombus in a left portal vein branch (Figure 1). There was no radiologic evidence of cirrhosis, mesenteric ischemia, acute diverticulitis, intra-abdominal abscesses, kidney stones, or gallstones. The remaining major intrabdominal organ systems were unremarkable. A few scattered small pulmonary nodules were noted in the lungs and thought to be inflammatory or infectious in nature. There was no focal consolidation in the lungs or pleural effusions. Finally, an enhancing endometrial mass was also discovered, believed to likely represent neoplasia, concerning for malignancy.

The patient was admitted to the hospital with the diagnosis of septic PVT and immediately started on IV heparin. General surgery was consulted for a possible thrombectomy, and it was felt that surgical options were not feasible at the time. Ultimately, the patient’s blood cultures grew gram-positive cocci, and she underwent an echocardiogram to rule out endocarditis. A repeat CT scan was performed on post-admission day four due to persistent and progressive abdominal pain, which revealed air within an inferior mesenteric vein tributary leading to an area of mild sigmoid diverticulitis.

The patient clinical status deteriorated throughout her course in hospital and required admission to the ICU. Despite aggressive treatment, the patient’s condition continued to decline, and she developed multi-organ failure. Her family decided to move forward with palliative care, and she died five days after her initial presentation to the hospital.

## Discussion

With a diagnosis of pylephlebitis, the primary clinical goals in the ED and ICU are to stabilize the patient and monitor for life-threatening complications such as overwhelming sepsis, portal hypertension, and intestinal ischemia leading to intestinal infarcts, perforation, and multi-organ failure. Although a rare pathology, we stress the importance of maintaining a high degree of clinical suspicion for PVT secondary to sepsis in patients with fever, abdominal pain, and nausea.

Diagnosis of PVT requires imaging in the form of a contrast-enhanced CT or MRI. Ultrasound may be used as an initial screening exam, but further cross-sectional imaging is necessary to determine the extent of the PVT and potential causes [1]. In cases of non-cirrhotic, non-malignant PVT where the etiology has yet to be determined, testing for hypercoagulable states, such as common myeloproliferative neoplasm mutations including JAK2 and V617F, can be considered [6].

In our patient’s case, the underlying etiology remains unclear due to the presence of multiple risk factors. One possibility is that the patient developed diverticulitis and subsequent bacteremia, resulting in the formation of a PVT, which became septic. There is also a potential role for the patient’s suspected endometrial malignancy, which could have induced a pro-coagulable state. While septic PVT is most commonly caused by *Bacteroides fragilis* or *Escherichia coli* [4], our patient’s blood cultures were positive for *Streptococcus constellatus*. Although a less common organism, this bacterium is part of the normal flora of the oral cavity, urogenital region, and intestinal tract, in keeping with an abdominal source of bacteremia. The potential of this being a contaminant cannot be fully ruled out, however, this patient's presentation was in keeping with sepsis.

Treatment of pylephlebitis

As in the case of other forms of sepsis, the mainstay of treatment for septic PVT includes early and appropriate antimicrobial therapy. Specifically, empiric broad-spectrum antibiotics should include agents that provide coverage of enteric facultative gram-negative bacilli, anaerobic species, and aerobic *Streptococcus* species. Piperacillin-tazobactam was the antibiotic selected for this patient. The duration of antibiotics is not well-established; however, a minimum of at least four weeks of antibiotics is recommended, up to six weeks if liver abscesses are present [7]. Additionally, the source of the infection should be identified promptly, and surgical intervention considered if warranted.

Historically, there has been controversy about the role of anti-coagulation in septic PVT, largely due to the lack of literature on this rare clinical presentation. Previously, expert opinion only recommended anticoagulation in the following situations: an acute and extensive thrombus, documented thrombus extension, persistent fever despite antibiotics or surgical intervention, presence of ischemia, clotting factor deficiency, or presence of neoplasm [8-9]. However, a recent retrospective study showed that anticoagulation significantly increases the rate of PVT resolution [5]. Furthermore, a literature review of 100 cases of pylephlebitis suggests that anticoagulation may decrease mortality [4].

## Conclusions

Septic PVT, otherwise known as pylephlebitis, is a rare condition and can be easily missed or misdiagnosed due to its vague presentation. To increase the detection of this pathology, early imaging should be considered for patients presenting with fever and abdominal pain to assess portal vein patency. Although previously controversial, recent evidence suggests that there is a role for initiating early anticoagulation for recanalization, which could reduce potential life-threatening sequelae associated with ischemia.
